# Supplementary figures and images for: Relationship between Decreased Mineral Intake Due to Oral Frailty and Bone Mineral Density: Findings from Shika Study
Source: Nutrients. 2021 Apr 5;13(4):1193. doi: 10.3390/nu13041193 (PMC8066385; doi:10.3390/nu13041193)

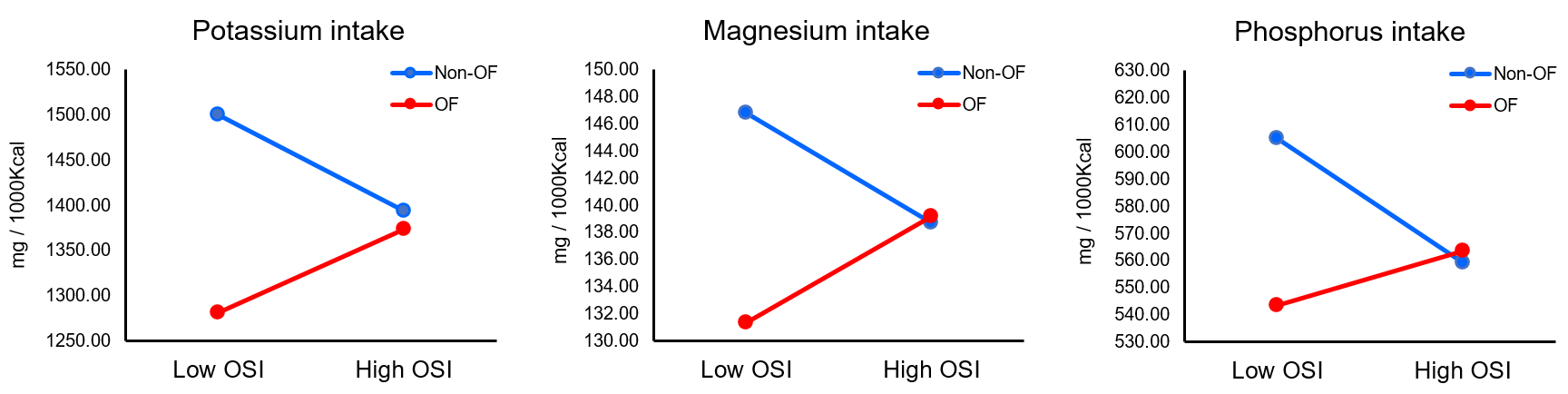

Supplement: Supplementary file 1 [file nutrients-13-01193-s001.zip › Figure S1.tif]

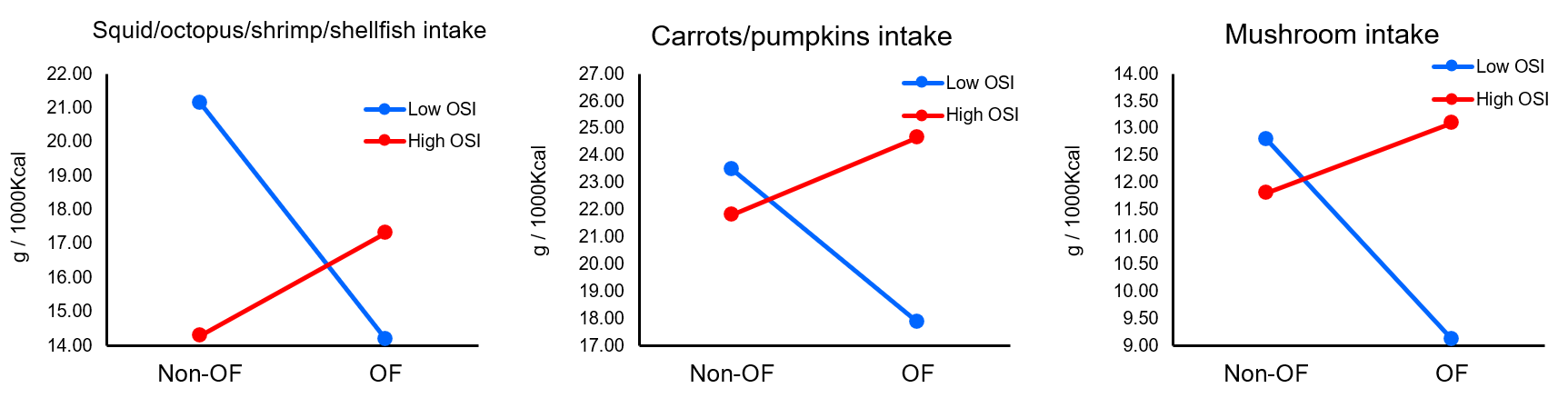

Supplement: Supplementary file 1 [file nutrients-13-01193-s001.zip › Figure S2.tif]

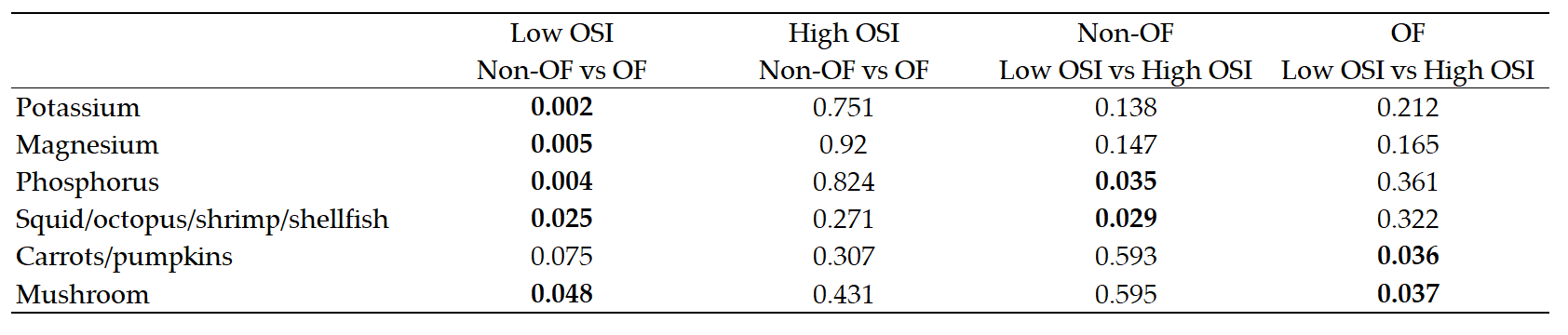

Supplement: Supplementary file 1 [file nutrients-13-01193-s001.zip › Revised Table S1.tif]
